# Dynamic Remodeling of Membranes and Their Lipids during Acute Hormone-Induced Steroidogenesis in MA-10 Mouse Leydig Tumor Cells

**DOI:** 10.3390/ijms22052554

**Published:** 2021-03-04

**Authors:** Sathvika Venugopal, Melanie Galano, Rachel Chan, Esha Sanyal, Leeyah Issop, Sunghoon Lee, Lorne Taylor, Pushwinder Kaur, Edward Daly, Vassilios Papadopoulos

**Affiliations:** 1 The Research Institute, McGill University Health Centre, Montreal, QC H4A 3J1, Canada; sathvika.venugopal@outlook.com (S.V.); rachel.ts.chan@gmail.com (R.C.); eshasanyal@students.aucmed.edu (E.S.); leeyah.issop@gmail.com (L.I.); sunghoon.lee2@mail.mcgill.ca (S.L.); proteomics.rimuhc@mcgill.ca (L.T.); pkaurpkaur@gmail.com (P.K.); edward.daly@mail.mcgill.ca (E.D.); 2 Department of Medicine, McGill University, Montreal, QC H4A 3J1, Canada; 3 Department of Pharmacology and Pharmaceutical Sciences, School of Pharmacy, University of Southern California, Los Angeles, CA 90089, USA; galano@usc.edu

**Keywords:** steroids, plasma membrane, mitochondria, endoplasmic reticulum, lipidomics, cholesterol, ATAD3A

## Abstract

Lipids play essential roles in numerous cellular processes, including membrane remodeling, signal transduction, the modulation of hormone activity, and steroidogenesis. We chose steroidogenic MA-10 mouse tumor Leydig cells to investigate subcellular lipid localization during steroidogenesis. Electron microscopy showed that cAMP stimulation increased associations between the plasma membrane (PM) and the endoplasmic reticulum (ER) and between the ER and mitochondria. cAMP stimulation also increased the movement of cholesterol from the PM compared to untreated cells, which was partially inhibited when ATPase family AAA-domain containing protein 3 A (ATAD3A), which functions in ER and mitochondria interactions, was knocked down. Mitochondria, ER, cytoplasm, PM, PM-associated membranes (PAMs), and mitochondria-associated membranes (MAMs) were isolated from control and hormone-stimulated cells. Lipidomic analyses revealed that each isolated compartment had a unique lipid composition, and the induction of steroidogenesis caused the significant remodeling of its lipidome. cAMP-induced changes in lipid composition included an increase in phosphatidylserine and cardiolipin levels in PAM and PM compartments, respectively; an increase in phosphatidylinositol in the ER, mitochondria, and MAMs; and a reorganization of phosphatidic acid, cholesterol ester, ceramide, and phosphatidylethanolamine. Abundant lipids, such as phosphatidylcholine, were not affected by hormone treatment. Our data suggested that PM–ER–mitochondria tethering may be involved in lipid trafficking between organelles and indicated that hormone-induced acute steroid production involves extensive organelle remodeling.

## 1. Introduction

Eukaryotic cells are comprised of fundamental structures known as organelles, most of which are compartmentalized within distinct membrane structures. These membranes create biochemical microenvironments that are specific to each organelle, and a number of essential biological processes take place at, or in, organelle membranes. Numerous studies over the past few decades have attributed the biological processes specific to each organelle to the distinct composition of proteins within the organelle membrane. For example, the plasma membrane (PM) is rich in receptor proteins, enabling both signal transduction and nutrient uptake [1], whereas the mitochondrial membrane contains proteins that function in electron transport system reactions and others that promote cholesterol trafficking for steroid biosynthesis [2]. Increasing evidence, however, suggests that, in addition to having distinct protein compositions, organelle membranes have their own unique lipid components [3]. These lipids also determine membrane properties and influence both membrane protein localization and function, which in turn, affects the role that each organelle plays in the cell [4,5,6]. As such, only a comprehensive understanding of the dynamic interaction between the lipidome and proteome for each organelle membrane can allow us to fully elucidate the biological function of any organelle.

Traditionally, lipid analyses were performed by extracting lipids from whole cells or tissues, followed by the separation and measurement of the lipid classes and their subspecies. However, this method eliminates all information about these lipids’ subcellular location, making it impossible to determine how lipid assemblies are organized. Therefore, to fully understand the biochemical reactions and pathways occurring at the subcellular level, it is essential to determine lipid localization and identify lipid assemblies by combining subcellular fractionation with the advanced mass spectrometry analysis of lipid species. Here, we used this strategy to perform the subcellular lipidomic analysis of cells involved in steroidogenesis to illuminate the dynamic changes in lipid profiles occurring in multiple organelles during the trafficking of cholesterol to the mitochondria for steroid biosynthesis.

Steroid hormones are essential for proper development, reproduction, and behavior, and their synthesis involves a well-regulated, multistep process that effectively meets physiological needs in a tissue-specific manner [7]. Mitochondria are considered to be the first sites for steroid synthesis. The stimulation of steroidogenic cells by pituitary hormones induces cyclic adenosine monophosphate (cAMP) production, which triggers the transfer of free cholesterol from intracellular stores that is destined to be converted into steroids to the outer mitochondrial membrane (OMM). Here, a multitude of cytosolic and mitochondrial proteins assemble to form a complex called the transduceosome. The transduceosome transfers cholesterol from the OMM to the inner mitochondrial membrane (IMM), where the cytochrome P450 side-chain cleavage enzyme, CYP11A1, with its cofactors, ferredoxin reductase (FdxR) and ferredoxin (Fdx), convert cholesterol to the first of the steroids, pregnenolone. Previous studies have shown that rapid protein synthesis, including the synthesis of some transduceosome proteins, is necessary for steroid production and that the inhibition of protein synthesis by the translation elongation inhibitor, cycloheximide, inhibits steroid formation [8,9].

During acute steroid biosynthesis, large quantities of cholesterol must be trafficked to the cholesterol-poor mitochondria. Though lipid droplets rich in cholesterol have been considered the classic source for mitochondrial steroid production, recent studies have focused on the endoplasmic reticulum (ER) as another possible cholesterol source [10]. The ER is a complex subcellular organelle running throughout the cell that performs several functions, including a broad array of lipid and protein biosynthesis reactions [11]. Though, like the mitochondria, the ER is poor in cholesterol, it is the site for de novo cholesterol synthesis. If not used for other means, this cholesterol, along with that which is obtained from circulating lipoproteins, will eventually be esterified and incorporated into the lipid droplets [7]. The ER also houses the cell’s sterol regulatory machinery, including the sterol regulatory element-binding protein (SREBP), which detects cholesterol levels in the ER and regulates the transcription of multiple genes involved in the synthesis of cholesterol and other fatty acids [12]. Recent electron microscopic studies have suggested that a direct organelle–organelle interaction occurs between the mitochondria and the ER upon hormonal stimulation in steroidogenic cells [10]. The associations between the two organelles are thought to be formed by microdomains rich in cholesterol that are known as mitochondria-associated membranes (MAMs) [13]. During acute steroid biosynthesis, these sites occupy about 5–20% of the mitochondrial surface and have been shown to possess unique lipid and protein compositions [9]. Interestingly, multiple transduceosome complex components are located in MAMs, suggesting that these also play a key role in cholesterol trafficking. 

The plasma membrane is another potential source of cholesterol for acute steroid biosynthesis. The first evidence of this role for the plasma membrane in steroid production came from studies by Freeman and colleagues [14,15,16,17,18]. They demonstrated cholesterol movement from the plasma membrane in multiple steroidogenic cell lines, including Leydig and adrenal cells. Another recent study using a fluorescently tagged domain 4 (D4) of the perfringolysin O (PFO) θ-toxin from *Clostridium perfringens*, which can bind cholesterol in membranes with a high affinity, showed an increased movement of cholesterol from the plasma membrane during hormonally stimulated steroidogenesis in mouse Leydig tumor (MA-10) cells [19]. Deng et al. extended these observations in in vitro reconstitution experiments demonstrating the vesicular trafficking of plasma membrane cholesterol for steroidogenesis in adrenal cortical cells [20], thus further validating a critical role for the plasma membrane in steroid biosynthesis. Based on these data, we and others have hypothesized that the plasma membrane may respond to hormonal stimulation by trafficking cholesterol to the ER, which in turn interacts with the mitochondria through MAM formation, delivering cholesterol via the steroidogenic metabolon to the IMM for steroid formation. This putative microdomain-mediated plasma membrane–ER–mitochondria association has been termed the plasma membrane-associated membrane (PAM). 

In this study, we quantified PAM interactions in MA-10 cells, which increased upon hormonal stimulation. We also found that hormonal stimulation induced the movement of fluorescently labeled cholesterol from the plasma membrane, which was inhibited when ATPase family AAA-domain containing protein 3 A (ATAD3A) was knocked down, suggesting that PAM interactions during steroidogenesis may be important for cholesterol trafficking from the plasma membrane to the mitochondria. We further analyzed the organization of three major lipid classes known to play essential roles in membrane structure and function in cells undergoing steroidogenesis. Specifically, we used MS analysis to measure lipids in isolated subcellular organelles in both PAM and MAM microdomains from MA-10 cells that were stimulated by basal and dibutyryl cyclic adenosine monophosphate (dbcAMP), a cell-permeable synthetic analog of cAMP, and in cells that were stimulated by dbcAMP in the presence of the steroidogenesis inhibitor, cycloheximide. These data revealed a dynamic reorganization of lipids in the membranes at the subcellular level upon the activation of steroidogenesis. They suggest that MAMs and PAMs play critical roles in lipid trafficking between organelles during this process. 

## 2. Results

### 2.1. Plasma Membrane–ER–Mitochondria Communication Increases with Dibutyryl Cyclic-AMP Treatment in MA-10 Cells

**Figure 1:**
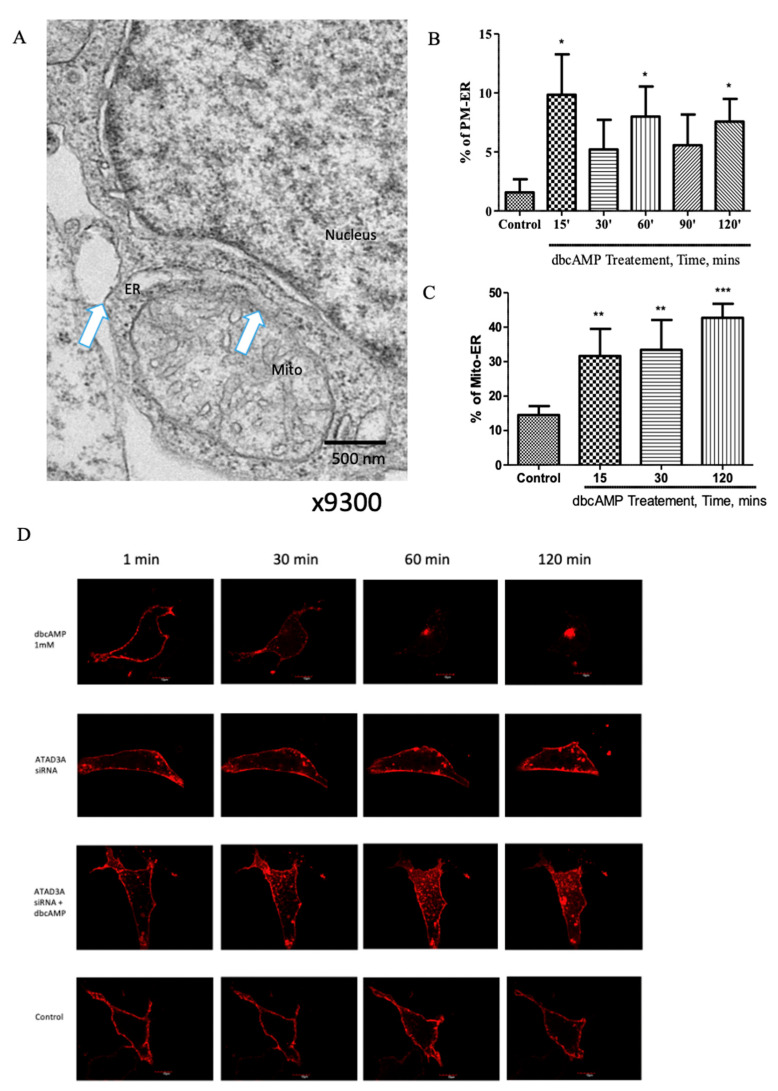

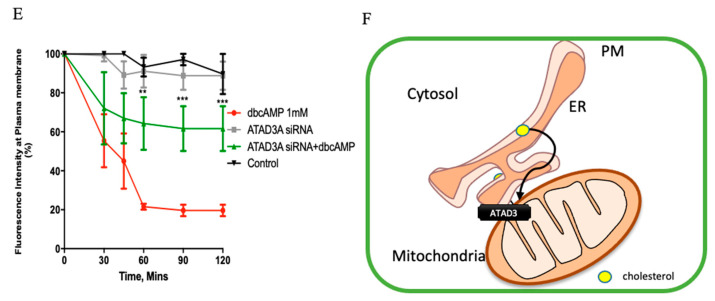
PM–ER–mitochondria interactions in control and hormone-stimulated MA-10 cells. (**A**) Contact site formation between the plasma membrane (PM), endoplasmic reticulum (ER), and mitochondria in MA-10 cells stimulated with 1-mM dibutyryl cyclic-AMP (dbcAMP), as visualized by electron microscopy (magnification: 9300x; scale bar: 500 nm). Blue arrows represent the PM–ER and ER–mitochondria interactions that form the PM-associated membrane (PAM) association. (**B**) Quantification of PM–ER interactions in control cells and in cells treated with dbcAMP for 15, 30, 60, 90, and 120 min, based on analysis of electron microscopy images. Images from about 30 MA-10 cells were analyzed from each treatment condition. (**C**) Quantification of ER–mitochondria interactions that form the mitochondria-associated membrane (MAM) in the same cells shown in (**B**) in control cells and in cells treated with dbcAMP for 15, 30, 60, 90, and 120 min based on the analysis of EM images. Data are displayed as a percentage of total ER (**B**) or mitochondria (**C**) assessed from about 30 MA-10 cells from each treatment condition. (**D**) Representative images of plasma membrane-associated mCherry-D4 fluorescence in the control and dbcAMP-treated cells treated with small interfering RNA (siRNA) targeting *Atad3a* at various time points. Scale bar: 10 μm. (**E**) Time course of PM-associated mCherry-D4 fluorescence in control and dbcAMP-treated cells treated with siRNA targeting *Atad3a*. (**F**) Representative model of PAM formation and the role of ATPase family AAA-domain containing protein 3 A (ATAD3A) in facilitating ER and mitochondrial membrane associations. Data are shown as mean ± SD of at least three independent experiments performed in triplicate. Two-way ANOVA, followed by Bonferroni’s post-hoc test (*) was used to calculate statistical significance. * *p* < 0.05; ** *p* < 0.01; *** *p* < 0.001.

To examine the associations between the plasma membrane, ER, and mitochondria that occur during steroidogenesis, we performed electron microscopic studies in MA-10 cells treated with or without dbcAMP, which mimics the action of cAMP and activates cAMP-dependent protein kinases [21], like the physiological hormone luteinizing hormone (LH), for 15, 30, 60, 90, and 120 min (Figure 1A–C). The white arrows in Figure 1A indicate plasma membrane–ER and ER–mitochondria associations, known as PAMs [22]. We observed a significantly increased interaction between the plasma membrane and ER within 15 min of treatment with 1-mM dbcAMP, followed by a stable increase in association for up to 120 min after stimulation compared to untreated control cells (Figure 1B). This positively correlated with an increased and progressive time-dependent association between the mitochondria and ER (Figure 1C), thus suggesting that MAMs and PAMs may play roles in trafficking cholesterol to the mitochondria during steroidogenesis.

ATAD3A is a key protein involved in forming MAMs and in the process of cholesterol trafficking into the mitochondria during steroid biosynthesis. Previous studies have shown that hormonal stimulation does not affect ATAD3A expression in MA-10 cells but does lead to increased MAM formation, and ATAD3A is essential for mitochondria–ER interactions [10]. Therefore, to further assess the role of MAMs in this process, we tracked the movement of free cholesterol during steroidogenesis in MA-10 cells subjected to the small interfering RNA (siRNA)-mediated knockdown of ATAD3A expression. This was accomplished by transfecting cells with an mCherry-D4 plasmid-expressing fluorescent D4 protein and tracking fluorescence using scanning confocal microscopy, as described previously [19]. We found that in untreated MA-10 cells, the fluorescent D4 protein is predominantly bound to the plasma membrane. When these cells were treated with dbcAMP, however, the fluorescence intensity at the plasma membrane began to decline within 30 min, continued to decrease over the next 30 min, and remained consistently low; conversely, there was minimal change in the untreated control cells (Figure 1D,E). Notably, in ATAD3A knockdown cells treated with 1-mM dbcAMP (Figure 1D,E), the rapid decline in mCherry-D4 fluorescence that occurred at the plasma membrane after treatment with dbcAMP was partially prevented, with a maximally significant effect observed at 60-min post-treatment. This indicated that cholesterol is retained in the plasma membrane under conditions of decreased ATAD3A. Thus, when there is less ATAD3A available for MAM formation, cholesterol movement from the plasma membrane is inhibited, suggesting that the PAM association may represent the route for free cholesterol trafficking from the plasma membrane to the mitochondria during steroidogenesis (Figure 1F). 

### 2.2. Isolation and Analysis of Subcellular Fractions in MA-10 Cells 

**Figure 2:**
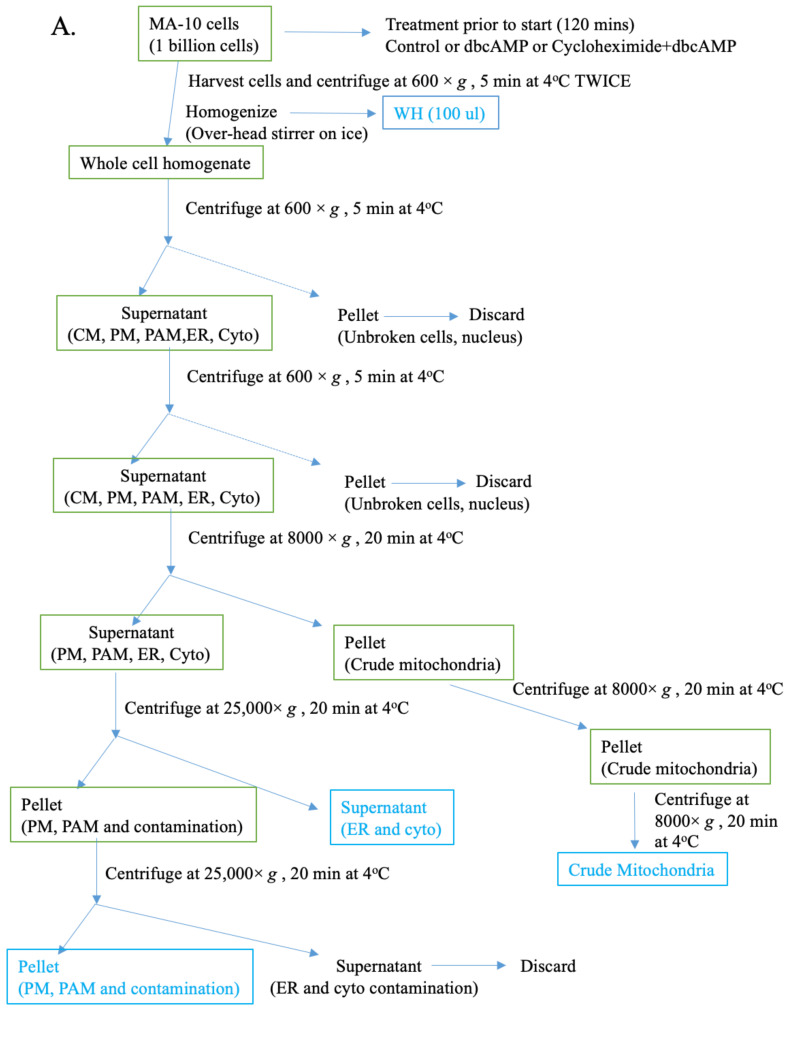

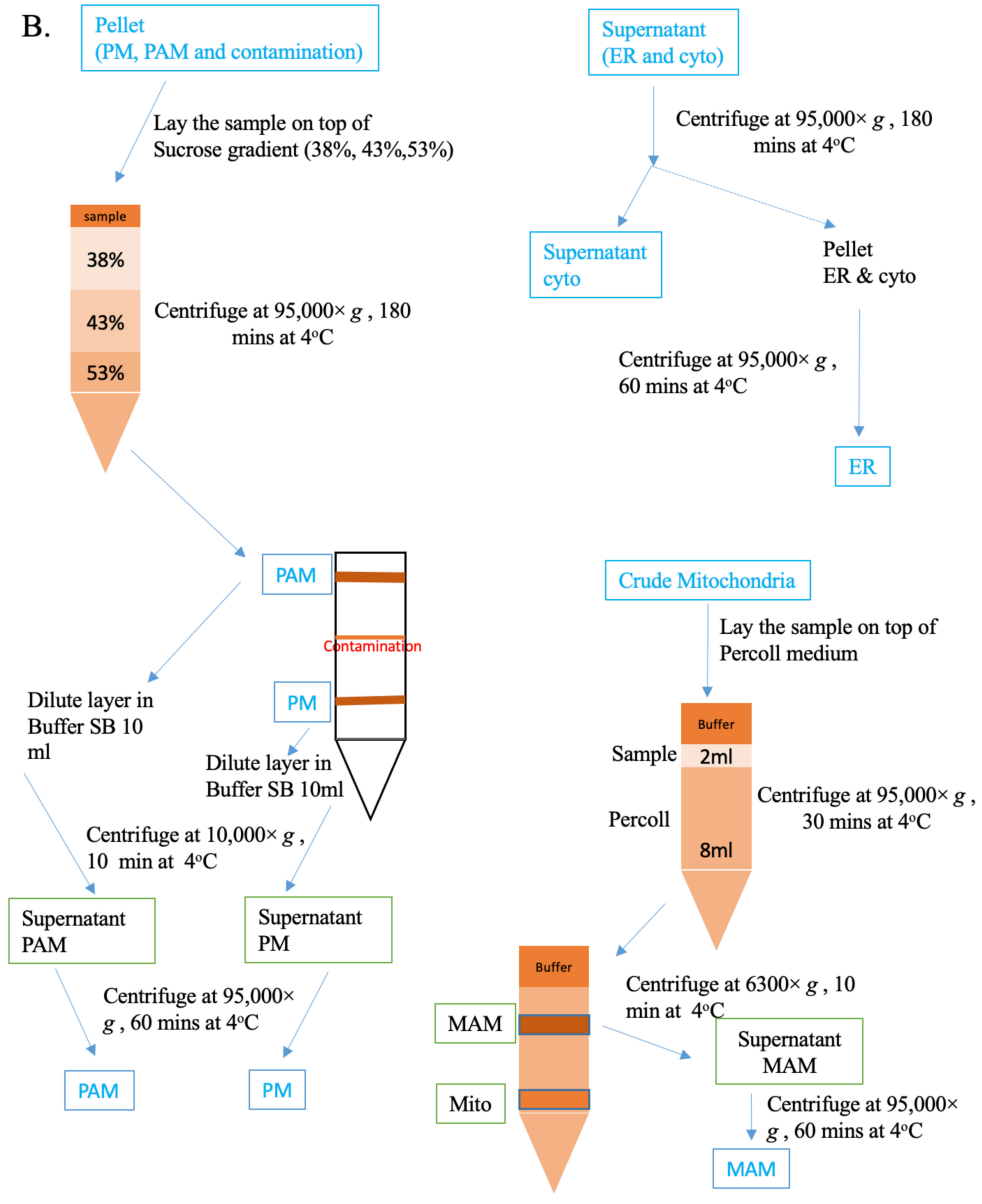

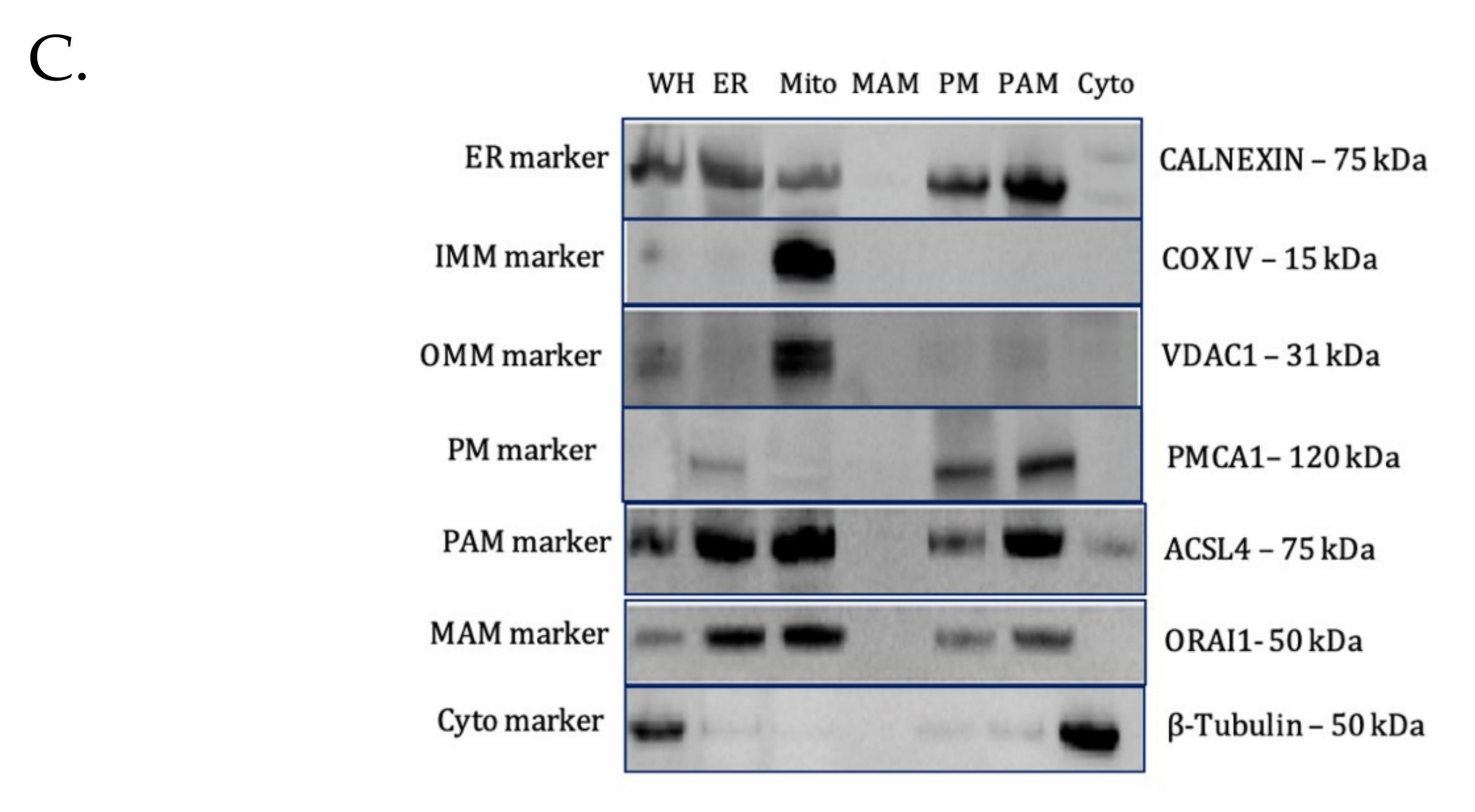
Subcellular fractionation and analysis of lipid enrichment. (**A**) Schematic representation of the protocol used for isolation of crude PM/PAM, ER/cytosol, and mitochondria/MAM fractions from MA-10 cells. (**B**) The crude PM/PAM fraction from (**A**) was further subjected to fractionation to generate pure PM, PAM, ER, cytosol, mitochondria, and MAM fractions. (**C**) Proteins from isolated organelles were subjected to immunoblot analysis to determine the purity of the samples by using antibodies to the following markers for the specified organelle: PM Ca(2+)-ATPase 1 (PMCA1) (120 kDa) for the PM, voltage-dependent anion channel (VDAC) for the outer mitochondrial membrane (OMM), β-tubulin for the cytoplasm, cytochrome c oxidase subunit IV (COX IV) for the inner mitochondrial membrane (IMM), calnexin for the ER, acyl-coenzyme synthetase long-chain family member 4 (ACSL4) for PAMs, and ORAI calcium release-activated calcium modulator 1 (ORAI1) for MAMs.

To investigate the distribution of cholesterol and other lipids in steroidogenic MA-10 cells, we first purified subcellular membrane fractions from untreated controls, as well as from cells treated with 1-mM dbcAMP, with or without 0.2-mM cycloheximide, for 2 h, using a modified technique that we developed by combining protocols previously published by Suski and colleagues and by Wieckowski and colleagues [22,23]. A schematic representation of the various differential centrifugation and gradient centrifugation steps involved in the isolation of the plasma membrane, ER, mitochondria, cytoplasm, PAM, and MAM fractions is shown in Figure 2A,B. For all subsequent analyses, cytoplasm fractions and whole cell homogenates were used as the negative and positive controls, respectively. To obtain quantities of the MAM and PAM fractions sufficient for protein estimation, immunoblot, and lipidomic analyses, large quantities of cells (~1 × 10^9^) were subjected to multiple differential centrifugation steps, as outlined in Figure 2A, to obtain the various crude fractions. Plasma membrane and PAM samples were further subjected to a sucrose gradient (38%, 43%, and 53%) ultracentrifugation, which allowed for the separation of PAMs from the plasma membrane (Figure 2B). Percoll gradient ultracentrifugation was also used to isolate MAMs from the mitochondria, and, by using supernatant from the first centrifugation series (Figure 2A), an additional ultracentrifugation step was performed to separate the ER from the cytoplasm (Figure 2B). 

Once isolated, the protein levels in each sample were measured by Bradford analysis, and the protein enrichment of each membrane fraction was assessed using immunoblot analysis (Figure 2C). For these experiments, we used calnexin as an ER marker, cytochrome c oxidase subunit IV (COX IV) as an IMM marker, voltage-dependent anion channel (VDAC1) as a mitochondrial marker, PM Ca(2+)-ATPase 1 (PMCA1) as a plasma membrane marker, acyl-coenzyme synthetase long-chain family member 4 (ACSL4) as a PAM marker, ORAI calcium release-activated calcium modulator 1 (ORAI1) as an MAM marker, and β-tubulin as a cytoplasmic marker. With the exception of ORAI1, these were all found to be enriched in their respective organelle fractions, thus confirming their purity and the efficiency of the used isolation procedure (Figure 2C). Only a limited amount of the MAM sample was obtained during isolation, which was the likely reason that we were unable to detect ORAI1 in our immunoblot analysis. 

### 2.3. Cholesterol Composition in Isolated Subcellular Membrane Fractions 

**Figure 3:**
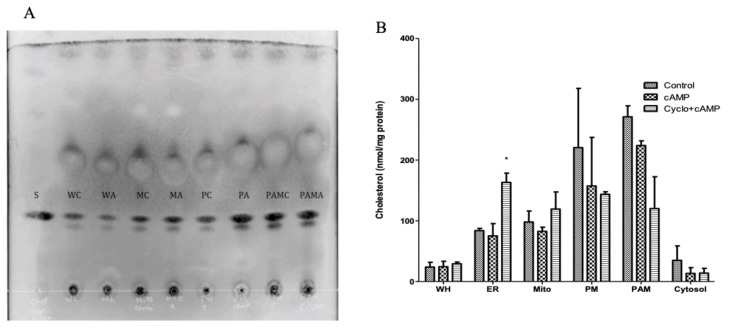
Cholesterol levels in subcellular fractions from MA-10 cells. (**A**) Lipids were isolated from organelle fractions. L: ladder; S: cholesterol standard; WC: whole cell lysate from control cells; WA: whole cell lysate from dbcAMP-treated cells; MC: isolated mitochondria from control cells; MA: isolated mitochondria from dbcAMP-treated cells; PC: isolated PM from control cells; PA: isolated PM from dbcAMP-treated cells; PAMC: isolated PAMs from control cells; PAMA: isolated PAMs from dbcAMP-treated cells. (**B**) Organelle cholesterol distribution was measured by LC–MS/MS in whole cell lysate (WH), endoplasmic reticulum (ER), mitochondria (Mito), plasma membrane (PM), plasma membrane-associated membrane (PAM), and cytosol fractions from the control, dbcAMP-treated, and dbcAMP plus cycloheximide-treated MA-10 cells. Data are shown as mean ± SEM; *n* = 2; * *p* < 0.05.

To assess the potential for communication between the plasma membrane and mitochondria via PAMs during cholesterol trafficking, subcellular fractions were isolated from control and dbcAMP-treated MA-10 cells, with and without cycloheximide, as described above. Protein-normalized samples were then subjected to lipid extraction and run on high-performance thin-layer liquid chromatography (HPTLC) silica plates to measure differences in cholesterol concentration in the control and treated cells (Figure 3A). This thin-layer chromatographic analysis showed that the plasma membrane and PAM samples contained the highest cholesterol content levels compared to other organelles. However, no significant changes in total cholesterol levels were observed in organellar membranes isolated from dbcAMP-treated cells vs. control cells, likely due to the method’s low sensitivity.

Because of its low sensitivity, HPTLC was used only to confirm the presence of cholesterol in all fractions rather than to compare amounts of cholesterol between the fractions. To address this question using a more sensitive technique, cholesterol content was measured in isolated fractions using MS. In agreement with results from the HPTLC analysis (Figure 3A), MS confirmed the plasma membrane and PAM samples to have the highest levels of cholesterol overall (Figure 3B). The ER fractions contained the next highest cholesterol content, followed by the mitochondria and cytoplasm. Furthermore, the total cholesterol concentrations (~20-nmol/mg protein) in whole cell homogenates from treated and untreated cells were the same, suggesting that MA-10 cells strive to maintain stable cholesterol levels even when steroid biosynthesis is increased by 100-fold during two-hour stimulation with dbcAMP [19] (Figure 3B). The cholesterol content in the ER was also unchanged in the control and dbcAMP-treated samples, whereas a significant increase was observed in the samples treated with dbcAMP and cycloheximide. This indicated that under conditions where cholesterol trafficking is induced by dbcAMP but transduceosome complex function at the OMM is blocked with cycloheximide cholesterol accumulates in the ER, suggesting the ER as a likely route for cholesterol trafficking to the mitochondria in steroidogenesis (Figure 3B). Furthermore, cholesterol concentrations were decreased in both the plasma membrane and PAM fractions upon hormonal stimulation, albeit not significantly, which may suggest a role for the plasma membrane and PAM in cholesterol trafficking. A similar decrease was also noted in the cytoplasm samples (Figure 3B). In the mitochondria, a slight increase in cholesterol levels was detected in cells subjected to stimulation with cycloheximide treatment versus those treated with dbcAMP alone, which again indicated that by inhibiting the transduceosome complex function, a pool of unprocessed cholesterol builds up at the mitochondria (Figure 3B). 

### 2.4. Analysis of Major Structural Lipids during Steroidogenesis 

We next performed lipidomic analysis, focusing on the three major categories of mammalian lipids that are important for membrane association or that play a role during steroidogenesis; that is, glycerophospholipids, sphingolipids, and cholesterol and its esters. For these experiments, MA-10 cells were treated with dbcAMP, with or without cycloheximide, or left untreated as a control, and then subcellular fractions were isolated as described above. We then measured glycerophospholipids, including phosphatidylcholine (PC), phosphatidylethanolamine (PE), phosphatidylserine (PS), phosphatidylinositol (PI), phosphatidic acid (PA), and cardiolipin (CL); sphingolipids, including sphingomyelin (SM) and ceramides (Cer); and neutral lipid cholesteryl esters (CEs) to assess the role of these lipids in membrane reorganization and steroidogenesis. We did not analyze other categories of lipids, including glycerolipids and fatty acyls, due to their extreme hydrophobic or relatively hydrophilic nature, respectively. The highly hydrophobic lipids are usually neutral, do not function in membrane reorganization, and are mostly confined to specialized organelles, such as lipid droplets. In contrast, relatively hydrophilic lipids may release and redistribute from original membranes during extensive subcellular fractionation. 

**Figure 4:**
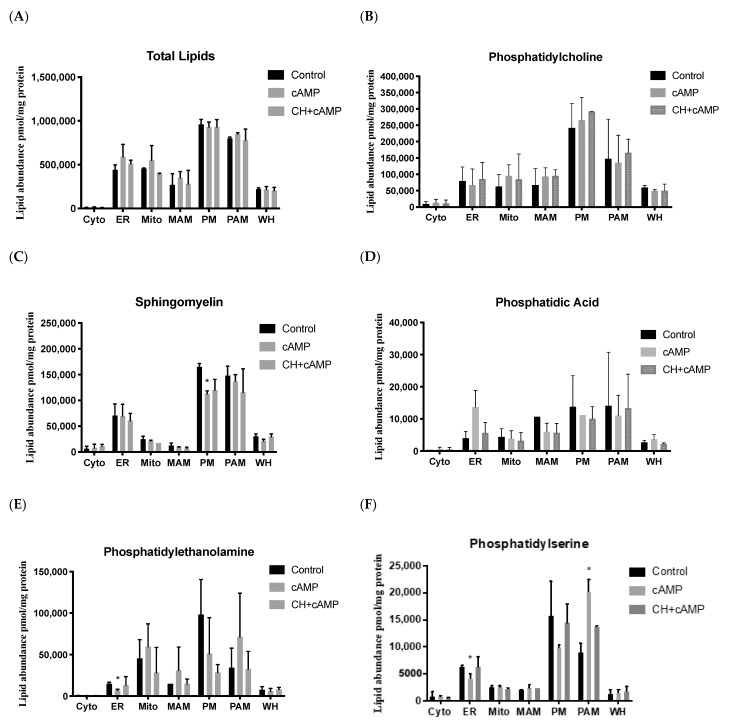

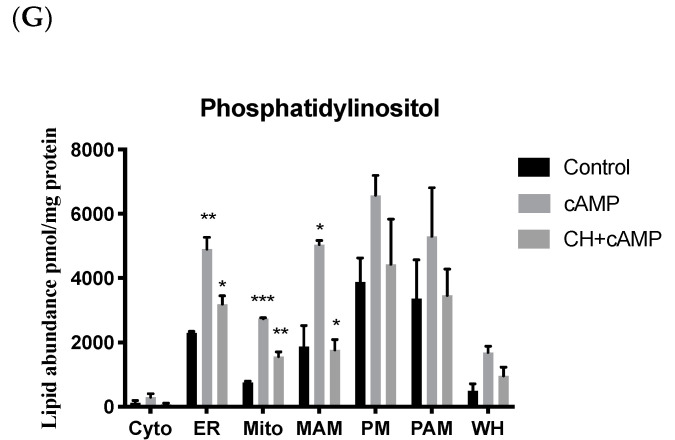
Lipid distribution profiles in subcellular fractions. Lipid abundance in cytosol (Cyto), endoplasmic reticulum, (ER), mitochondria (Mito), mitochondria-associated membrane (MAM), plasma membrane (PM), and plasma membrane-associated membrane (PAM) fractions, as well as whole cell extract (WC) from the control, dbcAMP-treated, and dbcAMP plus cycloheximide-treated MA-10 cells. (**A**) Total lipids, (**B**) phosphatidylcholine, (**C**) sphingomyelin, (**D**) phosphatidic acid, (**E**) phosphatidylethanolamine, (**F**) phosphatidylserine, and (**G**) phosphatidylinositol. Data shown are the average of results for each molecular species, mean ± SEM; *n* = 4; * *p* < 0.05; ** *p* < 0.01; *** *p* < 0.001.

A summary of all lipid species known to play essential roles in cholesterol trafficking and steroid biosynthesis that were analyzed in the six subcellular membrane fractions and whole-cell homogenates is presented in Table 1. In total, 221 lipid species were measured and compared in the control and treated samples. We found that the total lipid abundance and subcellular distribution were very similar between the different samples, with the plasma membrane having the highest lipid abundance, closely followed by the PAM fraction (Figure 4A). However, the overall lipid patterns observed were strikingly different in the plasma membrane and PAM fractions, substantiating previously published data showing that, although they are a part of the plasma membrane, PAM microdomains contain a different lipid profile that is indicative of their unique function [24]. The ER had the next highest overall abundance, followed by the MAM and mitochondria fractions. Though somewhat similar to one another, the PAM and MAM fractions also displayed distinct lipid profiles [25,26]. As expected, the cytoplasm samples showed a low lipid abundance (Figure 4A). 

In general, the lipid profiles of each fraction resembled the profile from whole-cell homogenates (Figure 4). The levels of phosphatidylcholine, the most abundant phospholipid class, were found to remain generally unchanged in corresponding fractions from each of the different treatments (Figure 4B and Figure S1), suggesting a fundamental role for this species in membrane organization and stability. A similar pattern was observed for the sphingolipids, for which no change was detected under different treatment conditions for each fraction, except for a significant decrease in the plasma membrane following hormonal stimulation (Figure 4C and Figure S2). Conversely, the levels of phosphatidic acid seemed to increase with hormonal stimulation in the ER, which is the site of phosphatidic acid production, and this was inhibited by treatment with cycloheximide (Figure 4D). In particular, multiple phosphatidic acid species were upregulated by dbcAMP stimulation, as detailed in Figure S4. 

For phosphatidylethanolamine, an important class of structural membrane lipids, we detected a notable increase in PAMs and MAMs from cells stimulated with dbcAMP. In contrast, a reduction in phosphatidylethanolamine abundance was observed in the plasma membrane fractions from cells treated with dbcAMP, and this redistribution of phosphatidylethanolamine from the plasma membrane to PAMs and MAMs was inhibited by cycloheximide treatment (Figure 4E and Figure S3). These data suggested an essential role for phosphatidylethanolamine in the formation of PAMs and MAMs upon hormonal stimulation (Figure 4E and Figure S3). However, the exact role of phosphatidylethanolamine in PAM and MAM formation requires further investigation. In the ER fraction, we saw a significant reduction in phosphatidylethanolamine upon hormonal stimulation. In mitochondria, a site of phosphatidylethanolamine synthesis, we detected an increased lipid abundance with hormonal stimulation inhibited by cycloheximide (Figure 4E and Figure S3). A similar finding was noted for the phosphatidylserine lipid class; we observed significantly decreased phosphatidylserine levels in the ER and significantly increased levels in PAMs, with a concurrent decrease in the plasma membrane samples upon dbcAMP treatment, suggesting the lateral movement of various phosphatidylserine and phosphatidylethanolamine lipid species from the plasma membrane to PAMs in response to hormonal stimulation (Figure 4E and Figure S3). 

Phosphatidylinositol has previously been shown to mediate acute responses to stimuli and participate in signal transduction [27]. Here, we found that this was the least abundant glycerophospholipid found in MA-10 cells (Figure 4G). Furthermore, in agreement with previously published data, we detected significant increases in phosphatidylinositol abundance upon dbcAMP stimulation in the ER, mitochondria, and MAM fractions. Notably, these increases were also significantly inhibited by cycloheximide in all fractions, suggesting that cycloheximide treatment exerts inhibitory effects on PI synthesis [28] (Figure 4E and Figure S5). 

### 2.5. DbcAMP-Induced Lipidome Remodeling

**Figure 5:**
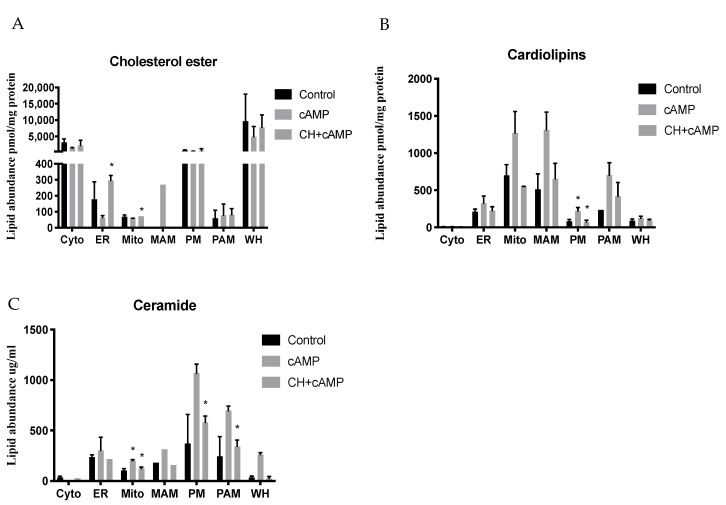

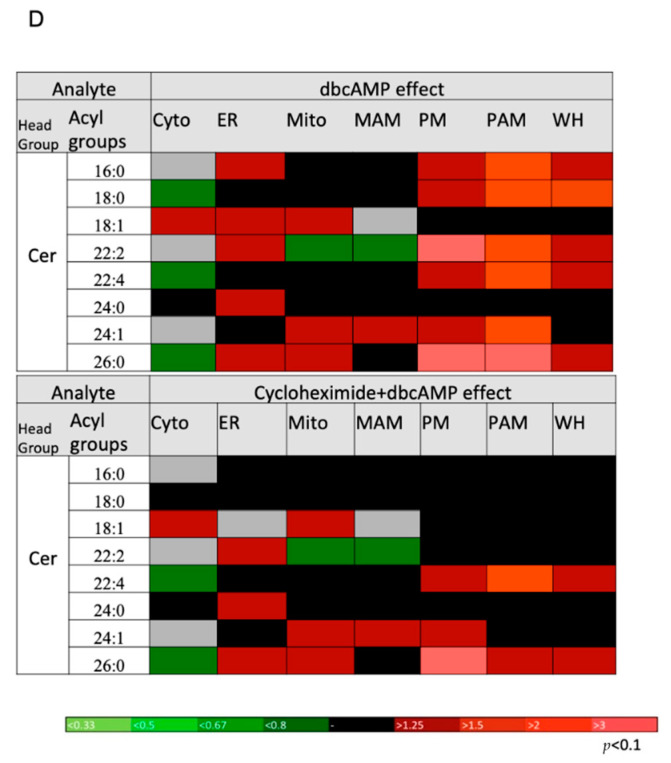
Cholesteryl ester, cardiolipin, and ceramide distribution profiles in subcellular fractions. (**A**) Cholesteryl ester, (**B**) cardiolipin, and (**C**) ceramide abundance in cytosol (Cyto), endoplasmic reticulum (ER), mitochondria (Mito), mitochondria-associated membrane (MAM), plasma membrane (PM), and plasma membrane-associated membrane (PAM) fractions, as well as whole cell extract (WC) from control, dbcAMP-treated, and dbcAMP plus cycloheximide-treated MA-10 cells. (**D**) Heat map analysis showing changes in ceramide (Cer) levels in response to stimulation with dbcAMP and dbcAMP plus cycloheximide in MA-10 cells. Acyl groups are abbreviated by the number of carbon atoms and double bonds per molecule. Changes are color coded according to the statistical significance table at the bottom of the figure. Data shown are the average of results for each molecular species, mean ± SEM; *n* = 4. * *p* < 0.05.

Cholesteryl esters are predominantly stored in cytosolic lipid droplets, but a small pool is also present in the plasma membrane [29]. Here, our data were consistent with previously published work, demonstrating that cytoplasm samples have the highest cholesteryl ester content, followed by the plasma membrane, ER, and mitochondria; the MAM and PAM samples contained negligible amounts of cholesteryl esters (Figure 5A and Figure S6). The whole-cell homogenate samples showed high cholesteryl ester levels, likely due to the presence of lipid droplets that were not isolated in the fractionation procedure (Figure 3A). Most differences in cholesteryl ester levels between the different treatment groups showed a consistent pattern in which the abundance drastically decreased with dbcAMP treatment (Figure 5A and Figure S6). Of the eight cholesteryl ester species measured, most displayed a significant decrease with dbcAMP treatment in all fractions, A notable exception occurred in the PAMs, however, where we detected an increase in cholesteryl ester species with dbcAMP treatment (Figure S6). 

Cardiolipins are primarily present in the mitochondria and have been previously shown to play an important role in the electron transport system and steroidogenesis [30,31]. We measured a total of 37 cardiolipin species in our lipidomics analysis and found that, as expected, the mitochondria and MAM fractions had the highest levels, followed by the ER fraction. We further noted a general increase in cardiolipins in these fractions with dbcAMP stimulation blocked by cycloheximide treatment, suggesting an important role of these species in steroid production. Interestingly, we also found a significant increase in cardiolipin levels in the plasma membrane fraction following hormonal stimulation, which was significantly inhibited by cycloheximide treatment (Figure 5B and Figure S7). 

Ceramides (Cer) are generally synthesized in the cell by the hydrolysis of sphingomyelin [32], and previous studies have shown these can significantly increase progesterone production in MA-10 Leydig cells [33]. Accordingly, we found a significant increase in ceramide levels in the mitochondria fraction, which was significantly inhibited by cycloheximide treatment (Figure 5C). We also noted an overall significant increase in five out of the eight ceramide species analyzed in whole-cell homogenates. These seem to be predominantly present in the plasma membrane, ER, MAMs, and PAMs, with increased levels observed in the dbcAMP-treated samples (Figure 5C,D). Specifically, we noted a significant increase in six out of the eight species analyzed in the plasma membrane and PAM fractions from dbcAMP-treated treated cells. Still, this increase was strongly inhibited by cycloheximide treatment (Figure 5C,D), suggesting that cycloheximide can inhibit the function of proteins involved in ceramide synthesis pathways. 

## 3. Discussion

Newly synthesized lipids and those obtained from extracellular sources are distributed throughout the cell by mechanisms that are not yet fully understood [34]. Even after decades of research, the fundamental question of how hydrophobic lipid molecules traverse through the aqueous cytosol has yet to be precisely answered. Several vesicular and non-vesicular lipid trafficking pathways have been proposed. However, the pathway by which large quantities of cholesterol are rapidly trafficked to the mitochondria across the aqueous milieu during acute and chronic steroid biosynthesis has not been well-defined. The classic vesicular trafficking methods are too slow, and so their kinetics do not support the urgent need for cholesterol during this process [35]. Soluble protein-mediated, non-vesicular lipid transfer pathways have also been suggested, and a few studies have shown the importance of such proteins, including steroidogenic acute regulatory protein (STAR), in steroidogenesis [36]. However, the kinetics of these pathways do not match the net amounts of cholesterol being trafficked. 

Alternatively, there is increasing evidence to suggest inter-organellar conduits as the primary route for cholesterol trafficking. Newly synthesized cholesterol and other lipids are quickly transported from the ER to other organelles such as the mitochondria and plasma membrane, causing the ER to have a loose organization of membrane lipids [31]. One pathway that has been indicated in cholesterol trafficking involves ER–mitochondrial tethering, known as MAM interactions. This mechanism would support the strict time constraints in which cholesterol needs to be available at the mitochondria. However, although the ER is the de novo cholesterol synthesis site, it contains only about 1–2% of the total cholesterol [31]. Since the ER and mitochondria are poor in cholesterol, this suggest the involvement of a third organelle for this mechanism to be operational. Accordingly, previous studies from our laboratory identified the plasma membrane as a potential source of cholesterol for this interaction [19]. In the present study, using electron microscopy, we observed increased plasma membrane–ER interaction within 15 min of dbcAMP stimulation, followed by an increased interaction between the ER and mitochondria in the same cells (Figure 1A–C). Additionally, the knockdown of ATAD3A, an MAM interaction protein, appeared to slow the movement of cholesterol from the plasma membrane (Figure 1D,E). Based on these observations, we propose that plasma membrane–ER–mitochondria tethering, also known as PAM interactions, constitutes the route for cholesterol trafficking during steroidogenesis (Figure 1F). 

MAM and PAM tethering have been shown to occur in specialized membrane regions, known as microdomains, similar to lipid rafts [37,38]. These microdomains are rich in lipids and specialized proteins that are important for their function [13]. Here, we showed that MAM and PAM interactions were significantly increased within 15 min after starting hormone stimulation in MA-10 cells (Figure 1A–C), suggesting a rapid reorganization of membranes and their lipids during steroidogenesis. Recent studies have also indicated that apart from cholesterol, these membrane–membrane interactions transport a number of other lipids, including sphingolipids, such as sphingomyelin and ceramide [39], and major phospholipids, including phosphatidic acid, phosphatidylserine, phosphatidylinositol, and phosphatidylethanolamine [27,40,41,42,43]. However, it is not yet understood how organelles tolerate such a massive redistribution of lipids during steroidogenesis and then maintain homeostasis. 

In the present study, we report a comprehensive subcellular lipidomic analysis of steroidogenic MA-10 Leydig cells during hormone stimulation. Specifically, we determined the abundance of major membrane lipids in cellular organelles and membrane interaction sites previously shown to be essential for steroidogenesis. For these experiments, we compared unstimulated control cells with those undergoing dbcAMP-induced acute steroidogenesis and dbcAMP-stimulated cells treated with the protein synthesis inhibitor, cycloheximide [44,45]. Previous studies have shown that although cycloheximide abolished the hormone- and cAMP-induced steroid production in primary rat and MA-10 mouse tumor Leydig cells, it did not affect basal steroid production [46,47]. Thus, we only examined the effect of cycloheximide on dbcAMP-treated cells. We then isolated plasma membrane, ER, mitochondria, and cytoplasm, MAM, and PAM fractions from cells in each treatment group, following the schematic representation in Figure 2A,B), and performed lipid profiling of these fractions, as well as whole-cell homogenates as a control. We verified our subcellular fractionation strategy by immunoblot analysis, which confirmed the purity of each fraction with the exception of the MAM sample, likely because our MAM yield was very low. Though we could not assure the purity of the MAM fraction, the immunoblot showed the presence of the MAM marker in the PAM fraction, and this assured the purity of the PAM fraction since MAMs contain microdomains that tether the plasma membrane, ER, and mitochondria. Our data showed that the lipid composition aligned with previously reported data. For example, the lipid profile of mitochondria under basal conditions consists of high levels of phosphatidylcholine, phosphatidylethanolamine, and cardiolipin, as well as low levels of sterols and sphingolipids, consistent with our results [31]. Due to the large number of cells required for these studies and the numerous steps needed to isolate the various organelles and membranes, we observed a lot of variability in the results. Though the trends of the seen changes are clear, not all data showed significance. 

Based on these experiments, we propose that the observed changes in lipid composition in various fractions after dbcAMP treatment represent the activated state of MA-10 cells. However, we note that the degree of change induced by dbcAMP treatment for a given lipid class may vary based on the abundance of the lipid itself. That is, low abundance lipids will show bigger fold changes, whereas the levels of highly abundant lipids may not change substantially within a membrane [48]. For example, cholesterol and phosphatidylcholine, shown in Figure 3B and Figure 4B, are highly abundant lipids essential for membrane integrity. Consequently, we did not expect significant differences in these lipids’ levels in fractions from any of the treatment groups. Surprisingly, however, we detected a significant increase in cholesterol in the ER fraction from MA-10 cells treated with dbcAMP and cycloheximide (induction of cholesterol trafficking but inhibition of transduceosome complex function). This strongly suggests that the route of cholesterol trafficking to the mitochondria transits through the ER (Figure 3B). Additionally, the slight but consistent decrease in cholesterol content observed in the plasma membrane and PAM samples after treatment with dbcAMP, plus or minus cycloheximide, indicated that the plasma membrane could be a cholesterol source during acute steroidogenesis and that the route of transport may include PAMs (Figure 3B). It is likely that cycloheximide treatment did not restore decreased cholesterol levels in the plasma membrane and PAM samples because cholesterol transport from the plasma membrane to the ER is not dependent on rapid protein synthesis, whereas cholesterol transport from the ER into the mitochondria necessitates the synthesis of specific transduceosome proteins, e.g., STAR [49]. 

Cholesteryl ester is a neutral lipid that is important for steroidogenesis and is predominantly stored in lipid droplets. For many years, it was assumed that lipid droplets were the sole source organelle for cholesterol during steroid production since one of the first studies to address this question showed a decrease in lipid droplet volume upon hormonal stimulation [50]. Critically, although lipid droplets could provide a constant supply of cholesterol to sustain steroidogenesis, this process’s kinetics do not support an acute hormonal response in cells. Thus, the decrease in cholesteryl esters observed in whole-cell homogenates and in cytoplasm fractions during stimulation (Figure 5A) may suggest the utilization of esters for steroid production. Still, these are unlikely to be the first responders to stimulation. 

The majority of glycerophospholipids are synthesized in the ER and the mitochondria [51,52]. The ER is also the main site for the production of phosphatidic acid, a class of lipids that act as a precursor for the biosynthesis of many glycerophospholipids, including cardiolipin [53]. Previous studies in preovulatory granulosa cells have shown increased phosphatidic acid formation upon hormonal stimulation [54], and, consistent with this, we detected a significant increase in phosphatidic acid levels in the ER fractions from stimulated cells, suggesting dbcAMP-induced de novo phosphatidic acid synthesis (Figure 4D). We also observed an increase in cardiolipin formation in the mitochondria of stimulated cells (Figure 5B), indicating that dbcAMP can also induce the mobilization of newly synthesized phosphatidic acid to the mitochondria, where it is translocated from the OMM to the IMM for cardiolipin production [55]. Thus, these data supported previous claims that dbcAMP can stimulate the formation of contact sites between the OMM and IMM during steroidogenesis [8]. 

MAM and PAM formation has been shown to play a substantial role in the transport of multiple lipids to and from the ER [13]. Phosphatidylethanolamine and phosphatidylserine are two such lipid classes that are synthesized and transported between the mitochondria and ER through MAM formation [56]. Phosphatidylethanolamine is synthesized in the mitochondria by the decarboxylation of phosphatidylserine [57], which is trafficked via MAMs. We found that stimulation with dbcAMP appeared to stimulate phosphatidylethanolamine accumulation in the mitochondria and MAM fractions, suggesting an increased synthesis of phosphatidylethanolamine in the mitochondria. This process would require phosphatidylserine to have been translocated from the ER (Figure 4E,F). Both phosphatidylethanolamine and phosphatidylserine also showed a similar trend. A reduction was detected in the plasma membrane fractions, with a concurrent increase in the PAM fractions, during cAMP stimulation, indicating the relocation of these lipids during PAM formation (Figure 4E,F). 

Phosphatidylinositol is another lipid class primarily synthesized in the ER and delivered to other membranes, particularly the plasma membrane (where it is predominantly located), by various pathways that are not entirely understood (Figure 4G) [27]. Phosphatidylinositol is involved in almost all events at the cell surface, including multiple signal transduction pathways. In rat adrenals, adrenocorticotropic hormone (ACTH) treatment induces a rapid increase in phosphatidylinositol, accompanied by a several-fold induction of corticosterone production [28]. In this same study, cycloheximide seemed to rapidly inhibit phosphatidylinositol, as well as steroidogenesis. In accordance with this, we noted an overall increase in phosphatidylinositol in the ER, mitochondria, MAM, plasma membrane, and PAM fractions upon dbcAMP stimulation, suggesting an essential role for phosphatidylinositol in steroid biosynthesis (Figure 4G). Notably, cycloheximide also seems to attenuate the synthesis of phosphatidylinositol and its function (Figure 4G). 

Previous studies in the adrenal cortex have shown a rapid activation of sphingolipid metabolism upon ACTH or cAMP stimulation [58]. This metabolism at the plasma membrane has also been correlated with cholesterol movement from the plasma membrane for steroid hormone secretion in Leydig testicular cells [59]. Sphingomyelin is hydrolyzed by sphingomyelinases, resulting in the formation of ceramide [39], a secondary messenger molecule involved in a variety of cellular events, including proliferation, stress response mediation, senescence, and cell cycle arrest. During dbcAMP-mediated steroidogenesis, ceramide levels were found to be increased, particularly in the mitochondria, plasma membrane, and PAM fractions, indicating the functional importance of this signaling lipid in steroid production (Figure 5C,D). Notably, several previous studies have suggested a negative role for Cer in steroidogenesis [60,61,62]. However, other studies have indicated that it is critical for lipid raft formation in the plasma membrane and could displace cholesterol from lipid umbrellas [63]. Here, the increase of ceramide detected in the plasma membrane and PAM fractions from cells stimulated with dbcAMP may suggest a role for ceramides in the formation of membrane interactions. 

As with all “omics” studies, our map of the dynamic lipidome during hormonal stimulation can only be used as an effective hypothesis building system. Critically, this initial analysis allows us to propose several testable hypotheses, establishing the framework for future studies that could provide a deeper understanding of the various aspects of lipid metabolism, membrane dynamics, and steroidogenic cellular physiology. Overall, the results from this study strongly suggest that the formation of MAM and PAM associations, and their essential roles in multiclass lipid trafficking between organelles, may be critical for maintaining cellular homeostasis during steroidogenesis. Therefore, this work represents a first step towards enhancing our understanding of the roles of membrane associations and dynamic subcellular lipid organization in cholesterol trafficking and steroidogenesis. 

## 4. Materials and Methods

### 4.1. Cell Culture 

Mouse tumor Leydig cells (MA-10) were kindly provided by Dr. M. Ascoli, University of Iowa, Iowa City, IA, USA. These were cultured with Dulbecco’s modified Eagle medium (DMEM)/Ham’s F-12 Nutrient Mixture (F12) (Invitrogen, Thermo Fisher Scientific, Waltham, MA, USA) with 1% penicillin and 1% streptomycin, and the culture was maintained at 37 °C. The MA-10 medium was supplemented with 5% fetal bovine serum and 2.5% heat-inactivated horse serum. For time-course experiments, cells were incubated with DMEM/F-12, without serum supplementation, in the presence of appropriate drug treatments.

### 4.2. Small Interfering RNA Transfection and Live Cell Imaging 

Cells were seeded onto 6-well plates at an initial concentration of 3 × 105 cells per well and immediately transfected using the TriFECTa Kit with DsiRNA duplex (Integrated DNA Technologies, Coralville, IA, USA) and Lipofectamine RNAiMAX Transfection Reagent (Life Technologies, Thermo Fisher Scientific). The following small interfering RNA (siRNA) duplexes (150 nM) were used for the knockdown of Atad3 (NM_179203): duplex 1, 5′-CCAUCGCAACAAGAAAUACCAAGAA-3′; duplex 2, 5′-CCAGUUUGACUAUGGAAAGAAAUGC-3′; and duplex 3, 5′-AGGACAAAUGGAGCAACUUCGACCC-3′. Gene expression and target gene knockdown were evaluated by qRT-PCR [10]. After 48 h, transfected cells were plated onto 35-mm diameter FluoroDish culture plates (World Precision Instruments, Sarasota, FL, USA) that had been pretreated with 0.1% gelatin and incubated at 37 °C for 24 h. At 75% confluence, cells were transiently transfected with 2 μg of mCherry-D4 plasmid, using 4 μL of a jetPRIME reagent (Polyplus transfection, Illkirch, France) and 200 μL of a jetPRIME buffer. Cells were visualized at room temperature using a FluoViewTM FV1000 scanning laser confocal microscope (Olympus Corp., Shinjuku, Japan) at 100× magnification, with an oil immersion objective (UPLSAP). Images were captured using the FluoView software (version 3.1) [64], and quantification and image processing were performed with Image-Pro Plus, version 6.3 [65], and ImageJ, version 1.47 (http://rsbweb.nih.gov/ij, accessed on 10 January 2021) [66]. Statistical analyses of the quantitative data were performed using Prism, version 5.0 (GraphPad Software, San Diego, CA, USA) [67].

### 4.3. Electron Microscopy

MA-10 cells cultured in 6-well plates were grown for 48 h and then treated with 1 mM dbcAMP for various lengths of time (0, 15, 30, 60, and 120 min). Treated cells were washed once in a sodium cacodylate buffer (0.1 M and pH 7.4) (Mecalab, Montreal, Quebec, Canada) and gently scraped to harvest. These were then centrifuged at 1500× *g* for 5 min, and the pellet was fixed with 2.5% glutaraldehyde (Sigma-Aldrich, St-Louis, MO, USA) overnight at 4 °C. Fixed cells were washed three times with dH2O and then stained with OsO4 1% Osmium (Mecalab, Montreal, QC, Canada) for 2 h at 4 °C. Stained cells were then dehydrated by incubation in increasing acetone concentrations ranging from 30 to 100%. These were subjected to infiltration with acetone-epon (mixed at varying ratios (1:1, 1:2, and 1:3 *v/v*)), the samples were embedded in pure epon (Mecalab, Montreal, Quebec, Canada), and polymerization was performed by incubation at 60 °C for 48 h. Blocks were cut into 100-nm slices, collected on 200 mesh copper grids (Electron Microscopy Sciences, Fort Washington, PA, USA), and post-stained with 4% uranyl acetate for 5 min and then with Reynold’s lead for 5 min. Cells on grids were observed with an FEI TECNAI 12 transmission electron microscope (Hillsboro, OR, USA), operated at 120 KV. Images were collected on a charge-coupled device (CCD) Camera (AMT XR 80 C) at the McGill University electron microscopy facility.

### 4.4. Isolation of Organelles, PAMs, and MAMs

PAMs, MAMs, plasma membranes, and mitochondria were isolated from MA-10 cells following the protocol published by Suski et al., with some modifications outlined in Figure 2 [22]. For these experiments, untreated controls and cells treated with 1 mM dbcAMP, with or without 0.2 mM cycloheximide (1 × 10^9^ cells from each group), were used as raw material for the multistep fractionation procedure. Cells were grown in 150 mm dishes to 80% confluence. These were then washed with phosphate-buffered saline (PBS) and incubated in serum-free media with or without 1 mM dbcAMP or 1 mM dbcAMP with 0.2 mM cycloheximide for 2 h. Cells were washed again with PBS, harvested using cell scrapers, and then centrifuged twice at 600× *g* for 5 min at 4 °C. Pellets were resuspended in 10 mL of ice-cold isolation buffer 1 (225 mM mannitol, 75 mM sucrose, 0.1 mM EDTA, and 30 mM Tris-HCl; pH 7.4), and the cells were homogenized using a Teflon pestle, attached to a motorized overhead stirrer, and run at 4500 rpm, with cell integrity checked every 25 strokes. Once 90% cell damage was observed, the homogenates were centrifuged twice at 600× *g* for 5 min at 4 °C. The supernatant was collected and centrifuged at 8000× *g* for 20 min at 4 °C. The resulting supernatant, containing the plasma membrane, PAM, microsomes, ER, and cytosolic proteins, was processed as described below.

The pellet, which contained the crude mitochondrial fraction, was resuspended and further subjected to 8000× *g* for 20 min at 4 °C centrifugation twice to eliminate excess contamination. The obtained pellet was resuspended in mitochondrial reaction buffer (250 mM mannitol, 5 mM HEPES (pH 7.4), and 0.5 mM EGTA) and layered onto a Percoll solution (225 mM mannitol, 25 mM HEPES (pH 7.4), 1 mM EGTA, and 30% Percoll (*v/v*)), followed by ultracentrifugation at 95,000× *g* for 30 min to yield crude MAM and mitochondria fractions. The samples were further purified by centrifugation at 6300× *g* for 10 min and further centrifuged at 95,000× *g* for 60 min, yielding pure MAM and mitochondrial fractions. The purity of the sample was verified using immunoblotting with antibodies to ACSL4 (Abcam, Cambridge, UK), COX IV (Santa Cruz Biotechnology, Dallas, TX, USA), and VDAC (Abcam, Cambridge, UK).

To isolate ER membranes from supernatant containing the plasma membrane, PAM, microsomes, ER, and cytosolic proteins, the sample was centrifuged at 25,000× *g* for 20 min at 4 °C. The supernatant containing the ER and cytosol was centrifuged at 95,000× *g* for 180 min, resulting in a pellet containing the ER and cytoplasm. This pellet was resuspended and centrifuged again at 95,000× *g* for 60 min at 4 °C, which resulted in the isolation of the ER (pellet) and cytoplasmic fractions (supernatant). ER membranes were resuspended in isolation buffer 1, and the purity was verified by immunoblotting with calnexin antibody (Abacm). The pellet containing the plasma membrane and PAM fractions was subjected to ultracentrifugation at 25,000× *g* for 20 min at 4 °C to eliminate further ER and cytosol contamination. The thus obtained pellet was resuspended and layered onto a 4 mL 38%, 4 mL 43%, and 3 mL of 53% sucrose gradient before being centrifuged at 95,000× *g* for 180 min at 4 °C. This step ensured the separation of the plasma membrane and PAM fractions that formed two different layers in the sucrose gradient. The plasma membrane layer and PAM layer was separately collected, diluted in a 10 mL SB buffer and centrifuged at 10,000× *g* for 10 min at 4 °C. The collected supernatants were further centrifuged at 95,000× *g* for 1 h at 4°C to obtain pure PAM and plasma membrane pellets that were resuspended in 50-μL isolation buffer 2 (225 mM mannitol, 75 mM sucrose, and 30 mM Tris-HCl; pH 7.4). The enrichment of the samples was verified by immunoblotting using antibodies to TRPC3/6/7 (Santa Cruz Biotechnology) for the plasma membrane fraction and both ACSL4 (Abcam, Cambridge, UK) and ORAI1 (Santa Cruz Biotechnology, Dallas, TX, USA) for the PAM fraction.

### 4.5. Lipid Extraction and Thin-Layer Chromatography

Lipids from isolated membranes were extracted using the Bligh–Dyer method [68]. Briefly, samples were diluted to 1 mL with NaCl (154 mM) and dissolved in chloroform-methanol, 1:2 (*v/v*). These were vortexed for 1 min and then kept at room temperature (RT) for 10 min. A 1:1 (*v/v*) mixture of chloroform–NaCl (154 mM) was then added to the samples, and they were vortexed and centrifuged at 1800 rpm for 5 min at RT. The bottom phase, containing the lipids, was extracted using a Pasteur pipette and dried by being subjected to nitrogen gas. These lipids were then resuspended in chloroform–methanol, 1:1 (*v/v*). Samples were loaded as a 0.8-cm band onto a chloroform–methanol, 1:1 (*v/v*), prewashed, and heat-activated (100 °C for 1 h) HPTLC plate (silica gel 60 10 × 10 cm, EMD Millipore Corporation, Burlington, MA, USA). Lipid standards, 10 μg of cholesteryl ester 17:0 and 10 μg of cholesterol (Avanti Polar Lipids, AL, USA), were added in separate lanes. The plate was developed in a hexane:diethyl ether:acetic acid (70:30:1) mobile phase and then sprayed with primuline stain (Sigma-Aldrich, St-Louis, MO, USA). Spots were visualized under UV light and quantified with a BAS 5000 image analyzer (Fuji Film Inc., Tokyo, Japan).

### 4.6. Cholesterol Analysis Using TOF-MS

To quantify cholesterol in subcellular organelles, fractions were first spiked with a d6-cholesterol internal standard. Samples were then extracted twice with 1 mL of methyl tert-butyl ether (MTBE). Aliquots were combined, dried in a Thermo Speed Vac™, re-dissolved in 500-µl aliquots of MTBE, vortexed, and dried again. Dried samples were re-suspended in 200-μL 50% (aq.) methanol, vortexed, and transferred into auto-sampler vials (Thermo Fisher Scientific, Waltham, MA, USA). The resolubilized samples were stored at –20 °C until ready for LC–MS analysis. The sample analysis was performed on an AB SCIEX (Framingham, MA, USA) Triple-TOF 5600+ Mass Spectrometer coupled with a Shimadzu (Kyoto, Kyoto Prefecture, Japan) Nexera XR UHPLC system. A binary mobile phase consisting of water with 0.1% formic acid (Mobile phase A) and acetonitrile with 0.1% formic acid (Mobile phase B) was utilized, and 2-µL aliquots of each sample were injected into the LC–MS. Analytes were chromatographically resolved by isocratic elution (90% (B) at 350 µL/min) on an Agilent Eclipse Plus C-8 analytical column (50 mm × 2.1 mm ID × 1.8 µm particle). The total run time was 10 min, and the mass spectrometer was operated in a positive HESI mode, with a vaporization temperature of 550 °C and a spray voltage of 5.5 kV. For quantification, a calibration curve was analyzed along with each batch of samples. After the acquisition, the data were analyzed using the MultiQuant™ software [69].

### 4.7. Lipidomic Analysis with Hybrid Quadrupole TOF-MS

Lipid extracts from subcellular fractions were analyzed by flow injection analysis using the MSMSall method [70]. Approximately 100 μL of each extract were diluted 10-fold in 5 mM ammonium acetate in chloroform:methanol, 1:2 (*v/v*) and delivered to the source by isocratic flow at 7 μL/min of methanol:isopropanol, 3:1 (*v/v*), with 5 mM ammonium acetate, using a Shimadzu Prominence XR UFPLC autosampler and LC pump (Shimadzu Corporation, Kyoto, Japan).

The positive mode ion spray voltage was set at 5000 V, and the negative mode ion spray voltage was set to −4000 V, with a declustering potential at 40 V and an ESI source operating temperature of 150 °C. An atmospheric-pressure chemical ionization (APCI) probe and inlet were connected to a calibrant pump that delivered mass calibration solution. Sequentially, 50 μL of each sample were injected twice to complete both a positive mode and a negative mode based on the instrument manufacturer’s instructions. Positive and negative ion MS and sequential precursor ion fragmentation acquisitions were performed on a TripleTOF™ 5600+ System (AB SCIEX), controlled with Analyst^®^ TF 1.5.1 software, with MS/MSALL mode activated to perform a series of product ion scans. A high resolution TOF MS1 scan was run from m/z 200 to 1200, at an accumulation time of 300 ms. This was immediately followed by 1000 product ion scans for 1000 precursors evenly spaced from m/z 200.051 to 1200.051 (at unit mass resolution on the quadrupole) measuring across an MS/MS mass range of 100–1500, accumulated for 300 ms each, and collected in order from low to high m/z. The total time to perform one MS/MSALL acquisition in positive or negative mode was 5.48 min, with system flushing and equilibration for an additional 6 min. Solvent blanks (50 μL) containing the running buffer were run in between samples. All acquired TOF-MS and MS/MSALL data were processed with the LipidView™ 1.1 software (SCIEX) [71].

### 4.8. Statistical Analysis

Data are shown as means ± SEM of at least three independent experiments performed in triplicate. GraphPad Prism (San Diego, CA, USA) was used for graphic presentation. Two-way ANOVA, followed by Bonferroni’s post-hoc test (*) and the Student *t-*test were used to calculate statistical significance—* *p* < 0.05; ** *p* < 0.01; *** *p* < 0.001.

## 5. Conclusions

Taken together, these results revealed that hormone-induced acute steroid hormone production involves a dynamic reorganization of membrane lipids and extensive organelle remodeling in MA-10 Leydig cells. The data also suggested that plasma membrane–ER–mitochondria tethering (PAM interactions) may compose a route for cholesterol movement and supply during the early phases of steroidogenesis.

## Figures and Tables

**Table 1:** Overview of subcellular lipidome.

	Cytosol			ER			Mito			MAM			PM			PAM			WH		
Lipid Categories	Ctl	cAMP	C+C	Ctl	cAMP	C+C	Ctl	cAMP	C+C	Ctl	cAMP	C+C	Ctl	cAMP	C+C	Ctl	cAMP	C+C	Ctl	cAMP	C+C
Glycerophospholipids	42	42	42	150	150	150	173	173	173	172	172	172	164	164	164	180	180	180	183	183	183
Sphingolipids	10	10	10	19	19	19	15	15	15	16	16	16	17	17	17	19	19	19	19	19	19
Sterol lipids	11	11	11	7	7	7	5	5	5	5	5	5	9	9	9	6	6	6	11	11	11
Ceramides	5	5	5	8	8	8	8	8	8	7	7	7	8	8	8	8	8	8	8	8	8
**Total**	**68**	**68**	**68**	**184**	**184**	**184**	**201**	**201**	**201**	**200**	**200**	**200**	**198**	**198**	**198**	**213**	**213**	**213**	**221**	**221**	**221**

Summary of all lipid species known to play essential roles in cholesterol trafficking and steroid biosynthesis that were analyzed in the six subcellular membrane fractions and whole-cell homogenates. Each box shows the number of lipid species detected and quantified in the specific subcellular fraction in control (Ctl), dbcAMP (cAMP), and dbcAMP with cycloheximide-treated (cAMP+CH; C+C) MA-10 cells.

## Data Availability

The data presented in this study are available in the article or supplementary material.

## Supplementary Materials

The following are available online at https://www.mdpi.com/1422-0067/22/5/2554/s1.

## Abbreviations

ACTH	adrenocorticotropic hormone
ACSL4	acyl-coenzyme synthetase long-chain family member 4
APCI	atmospheric-pressure chemical ionization
ATAD3A	ATPase family AAA-domain containing protein 3 A
cAMP	cyclic adenosine monophosphate
cAMP+ CH	dbcAMP with cycloheximide-treated
Cer	ceramides
CL	cardiolipin
COX IV	cytochrome c oxidase subunit IV
CTL	control
D4	domain 4
dbcAMP	dibutyryl cyclic-AMP
DMEM	Dulbecco’s modified Eagle medium
ER	endoplasmic reticulum
Fdx	ferredoxin
FdxR	ferredoxin reductase
F12	Ham’s F-12 Nutrient Mixture
HPTLC	high-performance thin-layer liquid chromatography
IMM	inner mitochondrial membrane
LC–MS	liquid chromatography-mass spectroscopy
MA-10	mouse Leydig tumor cells
MAM	mitochondria- associated membrane
MS	mass spectrometry
MTBE	methyl tert-butyl ether
OMM	outer mitochondrial membrane
ORAI1	ORAI Calcium Release-Activated Calcium Modulator 1
PA	phosphatidic acid
PAM	plasma membrane -associated membrane
PBS	phosphate-buffered saline
PC	phosphatidylcholine
PE	phosphatidylethanolamine
PFO	perfringolysin O
PI	phosphatidylinositol
PM	plasma membrane
PMCA1	PM Ca(2+)-ATPase 1
PS	phosphatidylserine
qRT-PCR	quantitative reverse transcriptase-PCR
RT	room temperature
siRNA	small interfering RNA
SM	sphingomyelin
SREBP	sterol regulatory element-binding protein
VDAC1	voltage-dependent anion channel
