# Supplementary material for: Dynamic Remodeling of Membranes and Their Lipids during Acute Hormone-Induced Steroidogenesis in MA-10 Mouse Leydig Tumor Cells
Source: Int J Mol Sci. 2021 Mar 4;22(5):2554. doi: 10.3390/ijms22052554 (PMC7961408; doi:10.3390/ijms22052554)
Supplement: Supplementary file 1 [file ijms-22-02554-s001.pdf]

SUPPLEMENTAL DATA

Dynamic remodeling of membranes and their lipids during acute hormone-induced steroidogenesis in MA-10 mouse Leydig tumor cells

Sathvika Venugopal[1,2], Melanie Galano[3], Rachel Chan[1], Esha Sanyal[1], Leeyah Issop[1,2], Sunghoon Lee[1], Lorne Taylor[1], Pushwinder Kaur[1], Edward Daly[1], and Vassilios Papadopoulos[1,2,3,*]

From the [1]Research Institute of the McGill University Health Centre, Montreal, Quebec, H4A 3J1, Canada; [2]Department of Medicine, McGill University, Montreal, Quebec, H4A 3J1, Canada; [3]Department of Pharmacology and Pharmaceutical Sciences, School of Pharmacy, University of Southern California, Los Angeles, CA 90089, USA

Supporting Information Figures: 7

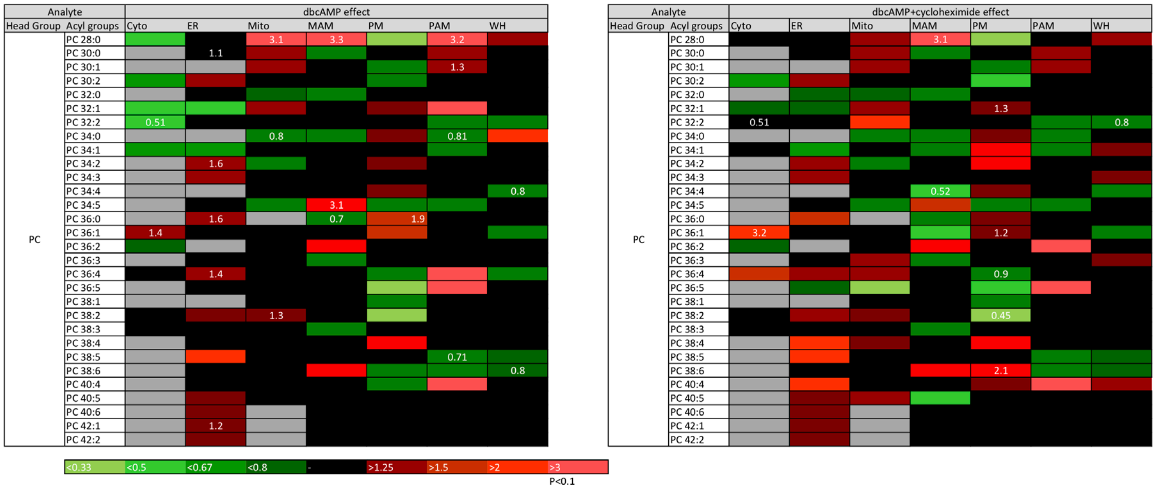

Fig S1. Phosphatidylcholine (PC) heat map analysis. Heat map analysis showing changes in PC levels in reponse to stimulation with dbcAMP and dbcAMP plus cycloheximide in MA-10 cells. Acyl groups are abbreviated by the number of carbon atoms and double bonds per molecule. Changes are color coded according to the statistical significance table at the bottom of the figure. Data shown are sums of results for each molecular species, mean ± standard error (SE), $n = 3$.

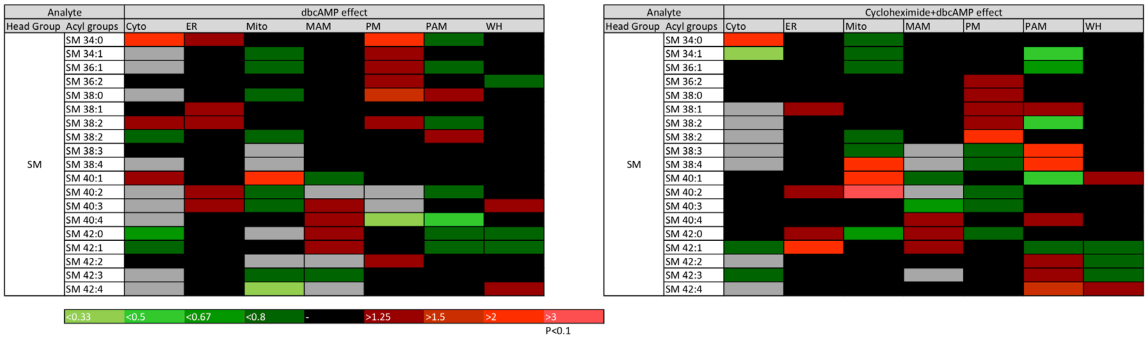

Fig S2. Sphingomyelin (SM) heat map analysis. Heat map analysis showing changes in SM levels in reponse to stimulation with dbcAMP and dbcAMP plus cycloheximide in MA-10 cells. Acyl groups are abbreviated by the number of carbon atoms and double bonds per molecule. Changes are color coded according to the statistical significance table at the bottom of the figure. Data shown are sums of results for each molecular species, mean ± SE, $n = 3$.

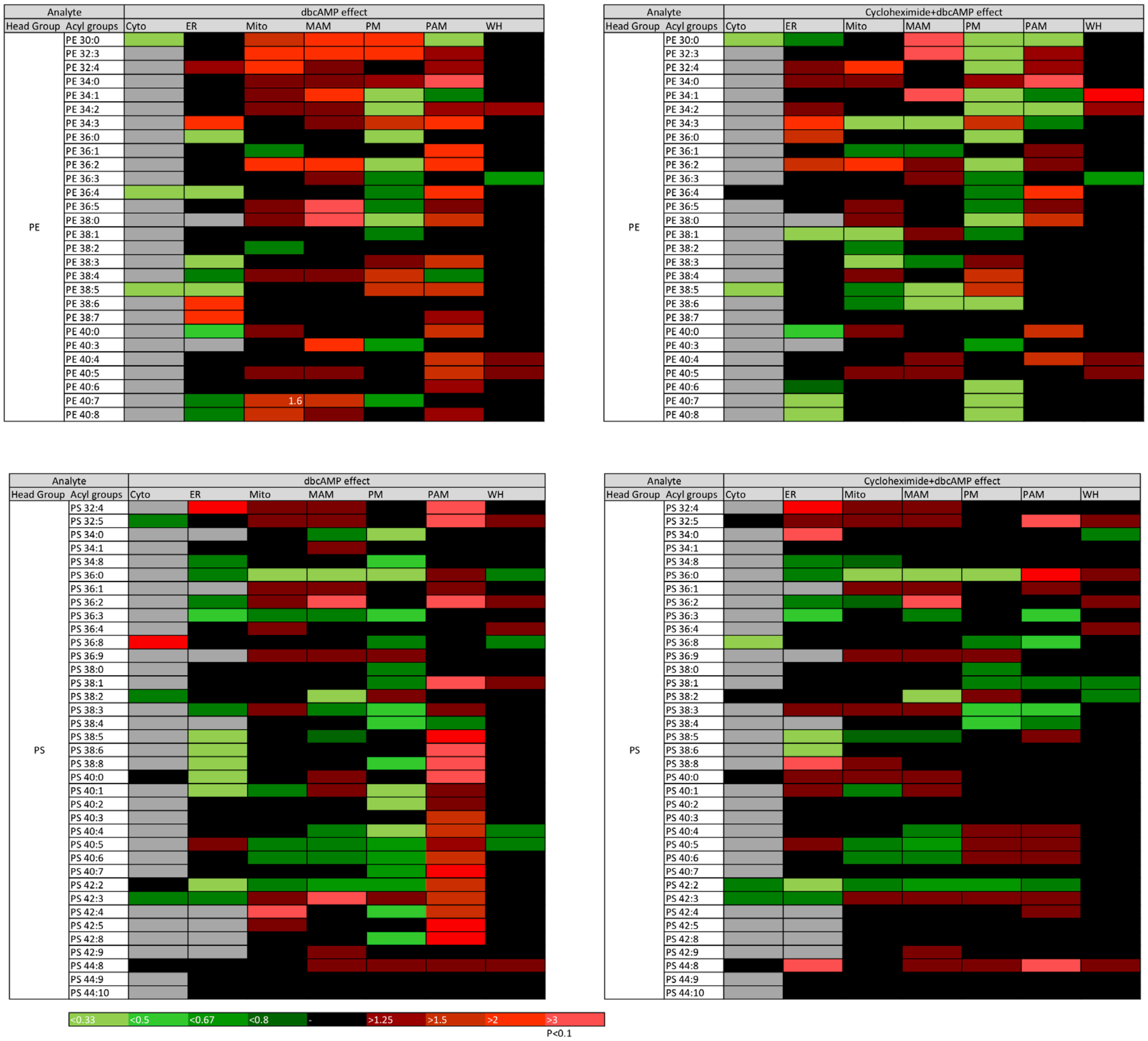

Fig S3. Phosphatidylethanolamine (PE) and phosphatidylserine (PS) heat map analysis. Heat map analysis showing changes in PE and PS levels in reponse to stimulation with dbcAMP and dbcAMP plus cycloheximide in MA-10 cells. Acyl groups are abbreviated by the number of carbon atoms and double bonds per molecule. Changes are color coded according to the statistical significance table at the bottom of the figure. Data shown are sums of results for each molecular species, mean ± SE, *n* = 3.

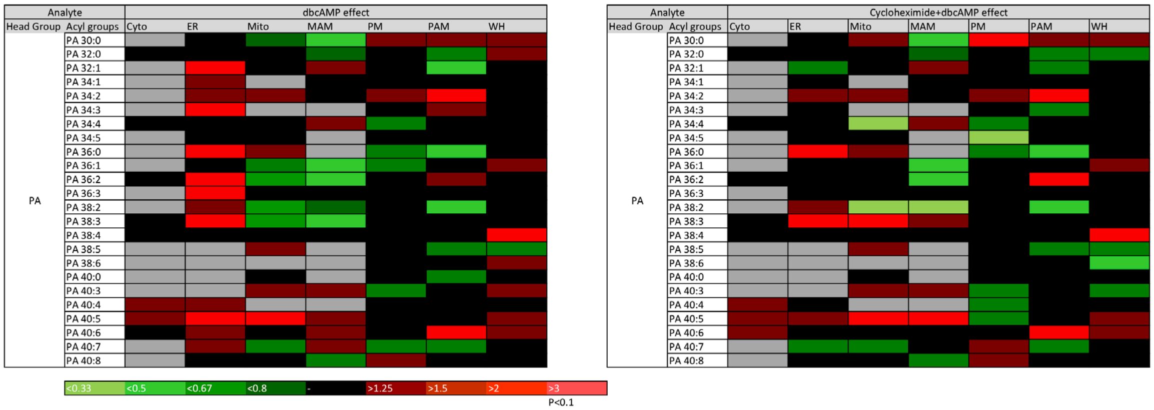

Fig S4. Phosphatidic acid (PA) heat map analysis. Heat map analysis showing changes in PA levels in reponse to stimulation with dbcAMP and dbcAMP plus cycloheximide in MA-10 cells. Acyl groups are abbreviated by the number of carbon atoms and double bonds per molecule. Changes are color coded according to the statistical significance table at the bottom of the figure. Data shown are sums of results for each molecular species, mean ± SE, $n = 3$.

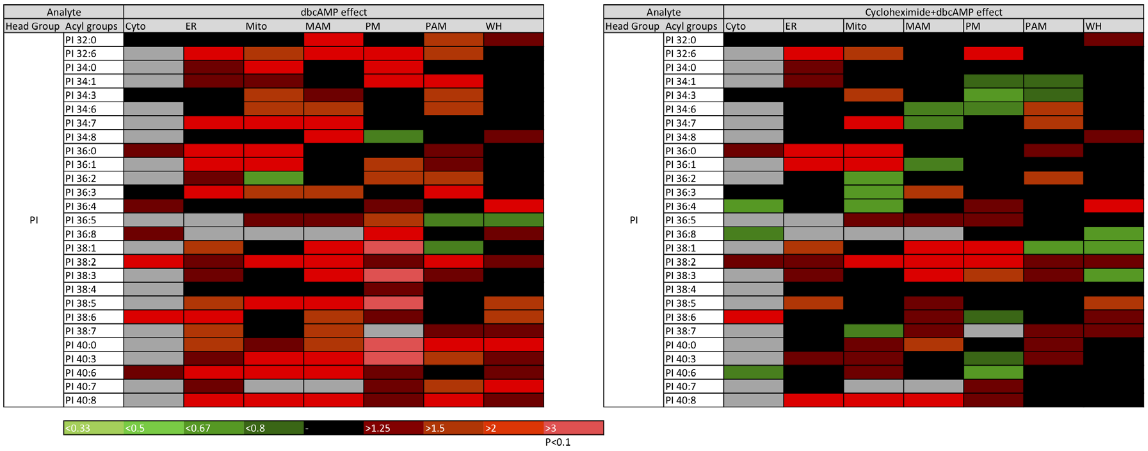

Fig S5. Phosphatidyl Inositol (PI) heat map analysis. Heat map analysis showing changes in PI levels in reponse to stimulation with dbcAMP and dbcAMP plus cycloheximide in MA-10 cells. Acyl groups are abbreviated by the number of carbon atoms and double bonds per molecule. Changes are color coded according to the statistical significance table at the bottom of the figure. Data shown are sums of results for each molecular species, mean ± SE, $n = 3$.

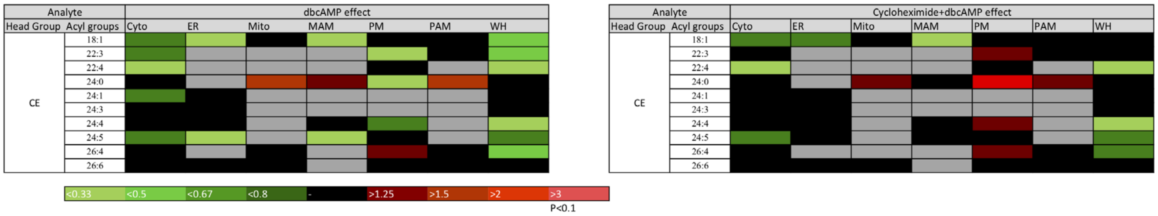

Fig S6. Cholesteryl ester (CE) heat map analysis. Heat map analysis showing changes in CE levels in reponse to stimulation with dbcAMP and dbcAMP plus cycloheximide in MA-10 cells. Acyl groups are abbreviated by the number of carbon atoms and double bonds per molecule. Changes are color coded according to the statistical significance table at the bottom of the figure. Data shown are sums of results for each molecular species, mean ± SE, *n* = 3.

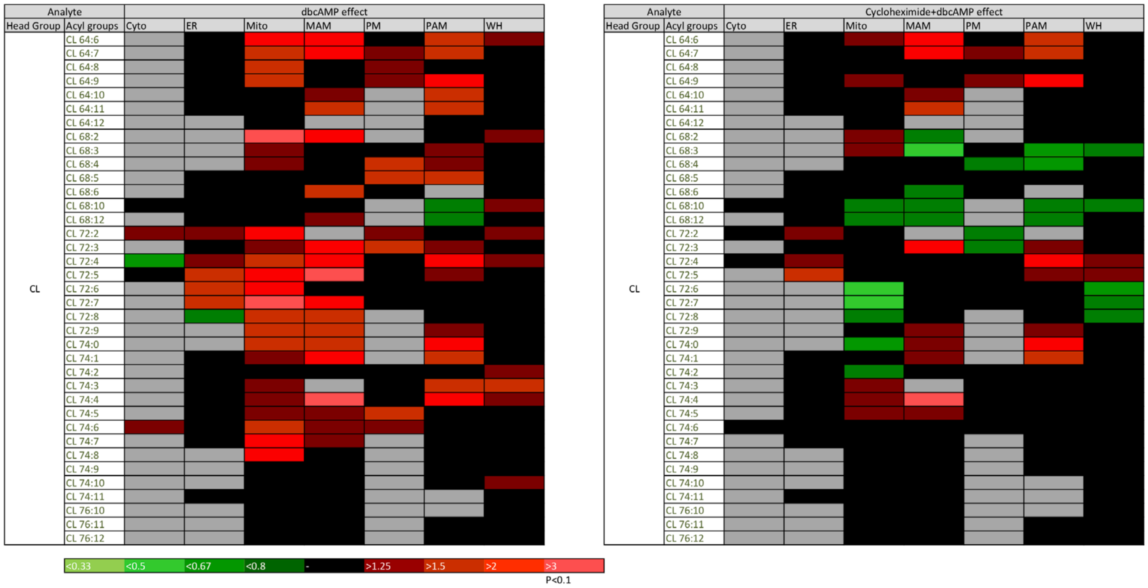

Fig S7. Cardiolipin (CL) heat map analysis. Heat map analysis showing changes in CL levels in reponse to stimulation with dbcAMP and dbcAMP plus cycloheximide in MA-10 cells. Acyl groups are abbreviated by the number of carbon atoms and double bonds per molecule. Changes are color coded according to the statistical significance table at the bottom of the figure. Data shown are sums of results for each molecular species, mean ± SE, $n = 3$
